# CD24-associated ceRNA network reveals prognostic biomarkers in breast carcinoma

**DOI:** 10.1038/s41598-022-25072-x

**Published:** 2023-03-07

**Authors:** Bin Yu, Ziyue Zhao, Zhuoyuan Chen, Cheng Xiang, Pingxiao Wang, Bo Xiao, Yu Xia, Aoyu Li, Tao Xiao, Hui Li

**Affiliations:** 1 Department of Orthopedics, The Second Xiangya Hospital, Central South University, 139th Renmin Middle Road, Changsha, Hunan China; 2 Orthopeic Biomedical Materials Engineering Laboratory of Hunan Province, 139th Renmin Middle Road, Changsha, Hunan China

**Keywords:** Cancer, Computational biology and bioinformatics, Immunology

## Abstract

Breast cancer is one of the most common cancer types which is described as the leading cause of cancer death in women. After competitive endogenous RNA (ceRNA) hypothesis was proposed, this triple regulatory network has been observed in various cancers, and increasing evidences reveal that ceRNA network plays a significant role in the migration, invasion, proliferation of cancer cells. In the current study, our target is to construct a CD24-associated ceRNA network, and to further identify key prognostic biomarkers in breast cancer. Using the transcriptom profiles from TCGA database, we performed a comprehensive analysis between CD24^high^ tumor samples and CD24^low^ tumor samples, and identified 132 DElncRNAs, 602 DEmRNAs and 26 DEmiRNAs. Through comprehensive analysis, RP1-228H13.5/miR-135a-5p/BEND3 and SIM2 were identified as key CD24-associated biomarkers, which exhibited highly significance with overall survival, immune microenvironment as well as clinical features. To sum up the above, the current study constructed a CD24-associated ceRNA network, and RP1-228H13.5/miR-135a-5p/BEND3 and SIM2 axis worked as a potential therapeutic target and a predictor for BRCA diagnosis and prognosis.

## Introduction

Breast cancer is one of the most common cancers worldwide, and makes up the largest proportion of cancer associated death in women. Over 2 million new female breast cancer cases were diagnosed and approximately 685,000 women died of breast cancer among 185 countries in 2020^1^. In addition, in 2022, the estimated number of new female breast cancer cases in the United States is up to 287850^2^. As a complex disease with genetic and clinical heterogeneity, breast cancer is generally categorized into 4 molecular subtypes, including Luminal A (ER+/PR+, HER2-, low proliferation), Luminal B (ER+/PR+, HER2-, high proliferation), HER2 enriched (HER2+, ER−, PR−; HER2+, ER+/PR+), Basal-like (HER2−, ER−, PR−) subtype^3–5^. Compared with other subtypes, luminal A presents a better clinical prognosis^4^. Luminal B subtype exhibits a lower expression of hormone receptors (ER and PR) but a higher expression of proliferation marker (Ki67) than Luminal A subtype^6^. HER2 enriched subtype has high sensitivity for anti-HER2 therapy^7^. Basal-like subtype, also known as triple-negative breast cancer (TNBC), is a highly aggressive tumor with poor prognosis, a high tendency to metastasis and recurrence, and less sensitivity to chemotherapy and radiotherapy^8,9^. What’s more, it was reported that multiple subtypes co-existed within a breast cancer^10^. The mortality of female breast cancer has decreased since 1989 due to early diagnosis and improvements in treatment methods^1^. The therapy strategy should be decided by molecular subtypes and locoregional tumor load. Generally, surgery, endocrine treatment, radiotherapy, neoadjuvant chemotherapy, and chemotherapy are basic therapy methods for breast cancer. Nowadays, breast conservation is technically feasible in many early breast cancers without metastasis, and the application of neoadjuvant chemotherapy extend the indication of breast conservation surgery^3,11,12^. In addition to the treatments mentioned above, some novel immunotherapy treatments are gradually adopted in clinical^13,14^, such as immune checkpoint blockades^15^, CAR-T cell treatment^16^, and mRNA vaccine technology^17^. Immunotherapy is a promising method for people with resistance to endocrine therapy or chemotherapy. For example, anti-PD-L1 therapy exhibited good efficacy in TNBC that lacks specific targets of endocrine therapy or chemotherapy but is rich in PD-L1^18,19^. Nonetheless, immunotherapy in breast cancer remains copious challenges. Some patients exhibited less insensitive to immunotherapy or resistant to immunotherapy after initial improvement^19^. Hence, it is crucial to explore the mechanism of immune regulation in breast cance. CD24 is a novel anti-cancer target which contributes to the immune escape and chemotherapy resistance in multiple tumors^20^. However, anti-CD24 therapy is controversial. CD24 is expressed not only in tumor cells but also in immune cells. CD24 is required for homeostatic proliferation of T cell, the blockage of which may contribute to the over-activation of proinflammatory reaction^20–22^. The role of CD24 in breast cancer is gaining increasing attention. It was reported that the subcellular translocation of CD24 was correlated with drug resistance in breast cancer^23^. In addition, CD24 is downregulated by ZBTB28, which enhances phagocytosis of macrophages and consequently inhibits breast cancer^24^.

Non-coding RNAs (ncRNAs), constitute the majority of the transcriptional products, and play significant roles in the regulatory of biological processes^25^. NcRNAs comprise circular RNA (circRNA), long non-coding NRA (lncNRA), microNRA (miRNA) and PIWI interacting RNA (piRNA), and their functions are variable^26^. Some ncRNAs can encode peptides or proteins^27^, whereas some can regulate the development of diseases via competing endogenous RNA (ceRNA) network^28^. CeRNA hypothesis proposes a post-transcriptional modulation that transcripts compete for translation via binding shared microRNAs^29^. In the past decades of years, ceRNA network has been identified involved in the initiation, development and drug resistance of various cancers^30–33^. In colorectal cancer, lncRNA CRART16 was found to facilitate expression of ERBB3 and induce cetuximab resistance via binding miR-371a-5p^34^. In human hepatocellular carcinoma, CD24 is regulated by MALAT1/miR-3064-5p/FOXA1 ceRNA axis, which consequently suppresses angiogenesis^35^.In breast cancer, ceRNA networks also play key roles. It was reported that tumor-derived exosome-mediated lncRNA SNHG16 induced CD73 + γδT cells in breast cancer samples from Chinese patients^36^. Regrettably, the ceRNA regulatory network associated with CD24 in breast cancer remains unclear. The studies for ceRNA network can provide us a novel insight into the pathological process of cancer, as well as help to dig out promising drug target or prognosis biomarkers. Therefore, we performed this study in order to identify significant ceRNA network in breast cancer.

## Materials and methods

### Data download and processing

**Figure 1:**
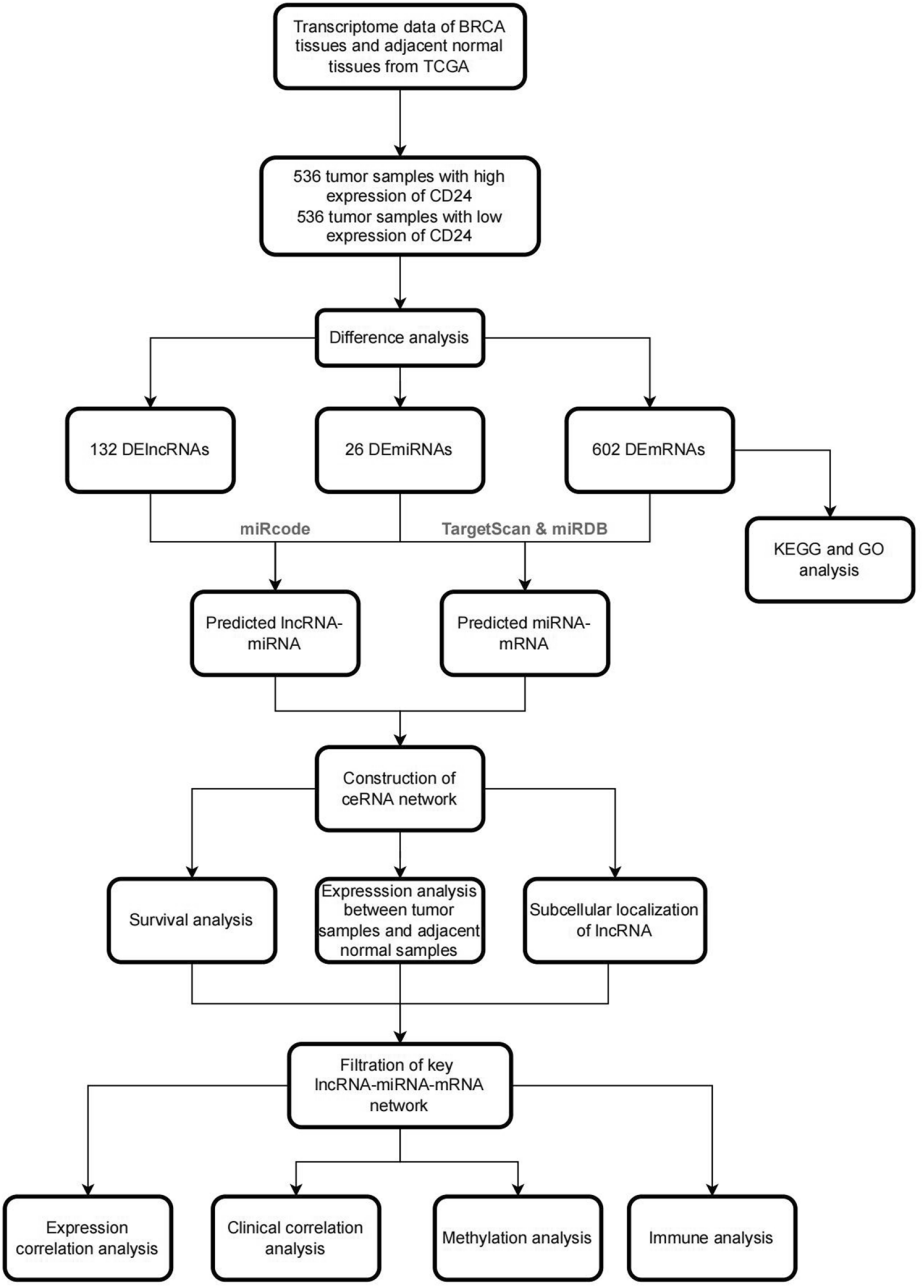
The flowchart of CD24 associated ceRNA network construction in breast invasive cancer (BRCA).

Our study processing was shown in Fig. 1. We downloaded transcriptional data and clinical information of breast invasive carcinoma (BRCA) from The Cancer Genome Atlas (TCGA) database (https://portal.gdc.cancer.gov/). Totally, gene expression raw data, miRNA expression raw data and clinical information for 1098 BRCA patients were downloaded. Finally, 1072 primary tumor sample were included in the study. In addition, 112 pairs of tumor samples and adjacent normal samples were extracted.

### Significant role of CD24 in breast cancer

First, our team obtained expression levels of CD24 in normal tissues through GTEx (https://www.gtexportal.org/). Then, we compared the expression level of CD24 between breast cancer samples and adjacent normal samples, and estimated its prognostic value through GEPIA web tool (http://gepia.cancer-pku.cn/). GTEx database contains gene expression data of 54 non-diseased tissues from nearly 1000 individuals^37^. GEPIA is a web tool, which provides various customizable functions to dig out significant genes, including differential expression analysis, pan-cancer analysis, survival analysis, correlation analysis and so on^38^.

### Screening of differentially expressed genes (DEGs)

To explore the role of CD24 underlying the pathogenesis of breast cancer, our study divided tumor samples into CD24^high^ tumor and CD24^low^ tumor using median expression level (read count = 42,946.5) as a criterion. Then, the current study performed a differential expression analysis between the two groups, and collected differentially expressed lncRNAs (DElncRNAs) with standard of *p* < 0.05 and |log2 fold change|> 0.3, differentially expressed miRNAs (DEmiRNAs) with standard of *p* < 0.05 and |log2 fold change|> 0.5 and differentially expressed mRNAs (DEmRNAs) with standard of *p* < 0.05 and |log2 fold change|> 0.7.

### Functional enrichment analysis of DEmRNAs

The R package “clusterprofile” was used to conduct the functional enrichment analysis of DEmRNAs through GO and KEGG database, with the restrictions of *p* < 0.05 and minimum count of 3. The GO enrichment result was visualized through “GOplot” package, whereas the KEGG pathway enrichment result was visualized by ggplot2 package.

### Construction of ceRNA network in BRCA and identification of hub RNAs

According to the hypothesis of ceRNA triple network, lncRNA is the first class of network and regulates the translation level of mRNAs via interacting with miRNA. Hence, the current study firstly explored lncRNA-miRNA pairs with high confidence via miRcode database (http://www.mircode.org). We then predicted mRNA targets of selected miRNAs using TargetScan (http://www.targetscan.org/) and miRDB (http://mirdb.org/), and extracted the same portion of their prediction results. The visualization of ceRNA network was created using Cytoscape 3.8.2, a visualized software used widely. The miRcode database predicts the interactions between lncRNAs and miRNAs based on seed complementarity and evolutionary conservation^39^. The targetScan database gets putative targets of miRNAs via matching the seed region of miRNAs and conserved 8mer, 7mer and 6mer sites of targets^40^. The miRDB database provides miRNAs targets that predicted by MirTarget tool, which was developed based on the interaction data of miRNA-target from high-throughput sequencing experiments^41^.

The filtration of hub genes integrated the results of survival analysis and lncRNA subcellular localization. LncRNA is a classical epigenetic regulative method and have a huge impact on the transcription of mRNA via interacting with miRNA. Hence, we explored hub lncRNAs first. We estimated prognostic value of DElncRNAs in ceRNA using logRank test and univariate Cox regression analyses. According to ceRNA hypothesis, the cellular location of lncRNA is highly correlated with its function. The lncRNA, localized in cytoplasm, can function as a sponge to regulate the expression of downstream genes. We downloaded the sequences of the prognosis associated lncRNAs from the LNCipedia database (https://lncipedia.org/)^42^, then we predicted the subcellular localization of lncRNAs via lncLocator web tool (http://www.csbio.sjtu.edu.cn/bioinf/lncLocator/)^43^. This web provided scores for subcellular location, including cytoplasm, cytosol, exosome, nucleus and ribosome, which reflected the possibility of localization of lncRNAs. In addition, we also analyzed the differences of tumor expression levels of key RNAs between tumor samples, normal samples, as well as paired adjacent normal samples in R software. Finally, we evaluated the correlations between key RNAs.

### Clinical correlation analysis

In order to further explore the significance of selected key genes in clinical application, the current study estimated the correlation between these genes and the following clinical features: age (< 35, 35–60, ≥ 60), clinical stage and TMN pathological stage.

### Immune infiltrate analysis of BEND3 and SIM2

The current study estimated the correlations between immune cell infiltration and hub mRNAs through TIMER database (https://cistrome.shinyapps.io/timer/) and TISIDB database (http://cis.hku.hk/TISIDB/index.php). Additionally, we also applied TISIDB to explore the relationships between immune checkpoint genes and hub mRNAs. TIMER is an online web application that provides researchers with correlation analysis between various immune cells and any gene of interest^44^. TISIDB provides a web resource for exploring and visualizing the interactions between immune system and genes^45^.

### Methylation analysis of BEND3 and SIM2

The current study applied three online web tools to estimate the methylation levels of BEND3 and SIM2, including UALCAN database (http://ualcan.path.uab.edu/), MEXPRESS database (https://www.mexpress.be/) and MethSurv database (https://biit.cs.ut.ee/methsurv/). UALCAN database provides access to public cancer data, and allows researchers to explore and visualize the correlation of gene expression and promoter methylation^46^. This database applies beta value to indicate promoter methylation level. Beta value 0.7–0.5 means hypermethylation whereas 0.3–0.25 means hypomethylation^47^. MEXPRESS is an online tool to analysis and visualize correlation between DNA methylation and clinical information based on TCGA public data^48,49^. MethSurv is a web tool providing information of single CpG^50^.

### Statistical analysis

The current study performed total statistical analysis via R software version 4.0.3. We performed difference analysis through “DEseq” R package, and performed survival analysis via “survival” R package. The expression levels of hub genes between tumor samples, normal samples and paired adjacent normal samples were estimated by Wilcoxon test. The correlations between hub ceRNA network members were identified by Pearson test. Wilcoxon test was used to compare the expression levels of hub genes between non-metastasis and metastasis groups, and Kruskal–Wallis test was used to estimate the expression differences of hub genes in clinical stage, degree of spread to regional lymph nodes, tumor size and age. *P < 0.05, **P < 0.01, ***P < 0.001.

## Result

### The tumor promoter role and clinical value of CD24 overexpression in BRCA

**Figure 2:**
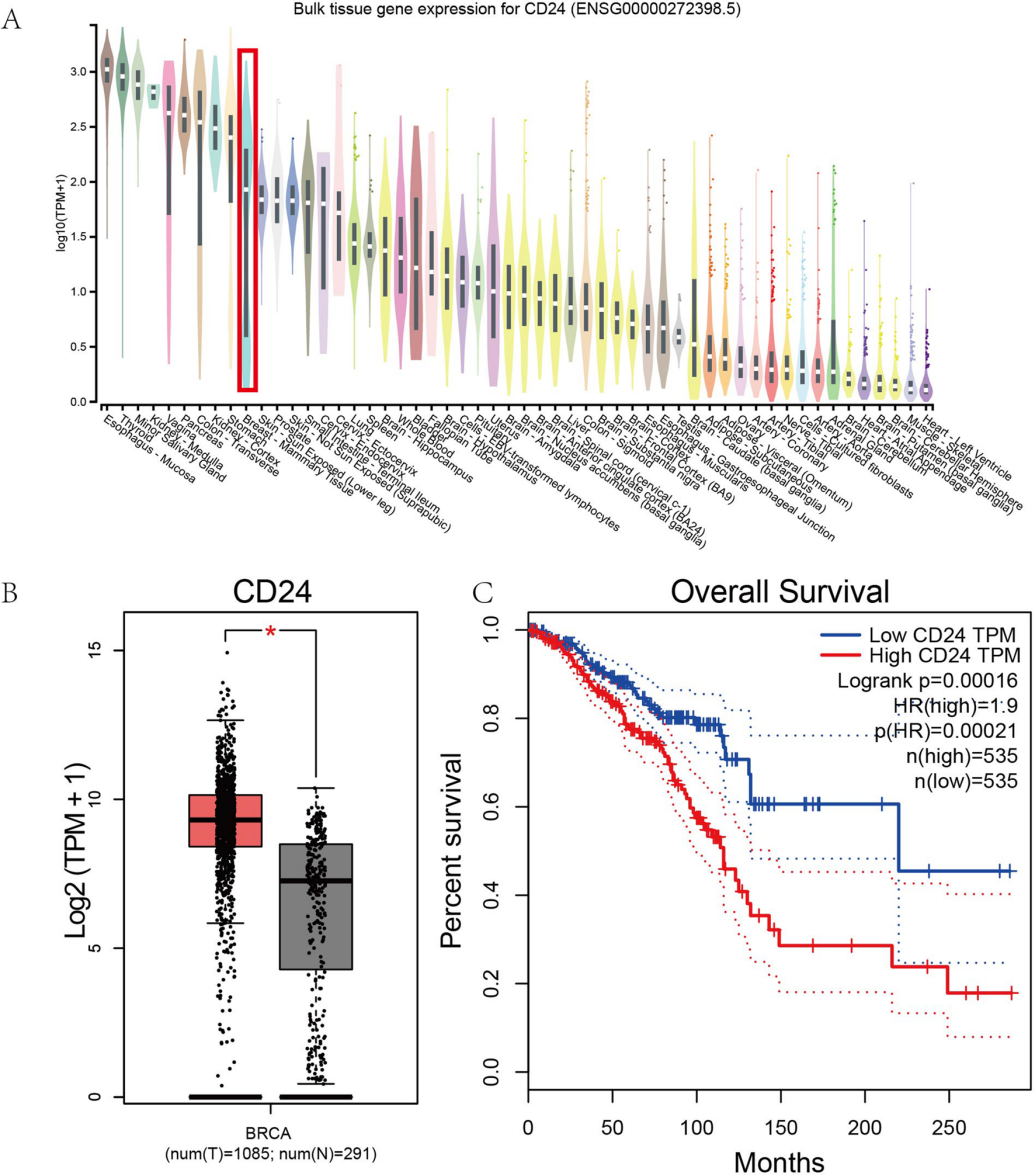
The function analysis of CD24 in BRCA. (**A**) Expression distribution of CD24 in various normal tissues using GTEx database. (**B**) Expression levels of CD24 in BRCA and normal tissues using GEPIA database. (**C**). Survival analysis of CD24 using GEPIA database.

In order to investigate the potential role of CD24 in BRCA, the current study explore the RNA expression of CD24 in normal breast tissue and BRCA tissue. We found that CD24 was highly expressed in breast mammary tissues based on GTEx database (Fig. 2A). Furthermore, based on GEPIA database, compared with normal breast tissues, CD24 was found upregulated in BRCA tissues (Fig. 2B).

Given that CD24 was abnormally overexpressed in BRCA tissues, the current study then explored the clinical values of CD24 expression in BRCA patients. The Kaplan–Meier survival curve (Fig. 2C) from GEPIA database showed that higher RNA expression of CD24 was correlated with poor prognostic result (Logrank test, *p* = 0.00016), and its HR score (HR = 1.9, *p* = 0.00021) revealed that CD24 served as a risk factor in BRCA.

### The identification of DEGs in BRCA

Our above analyses shed a light that CD24 was a key regulatory in BRCA, and the ceRNA network associated with CD24 had a potential to work as prognostic model for BRCA patients. Hence, the current study downloaded RNA sequence data and miRNA data of BRCA from TCGA database and performed further research.

**Figure 3:**
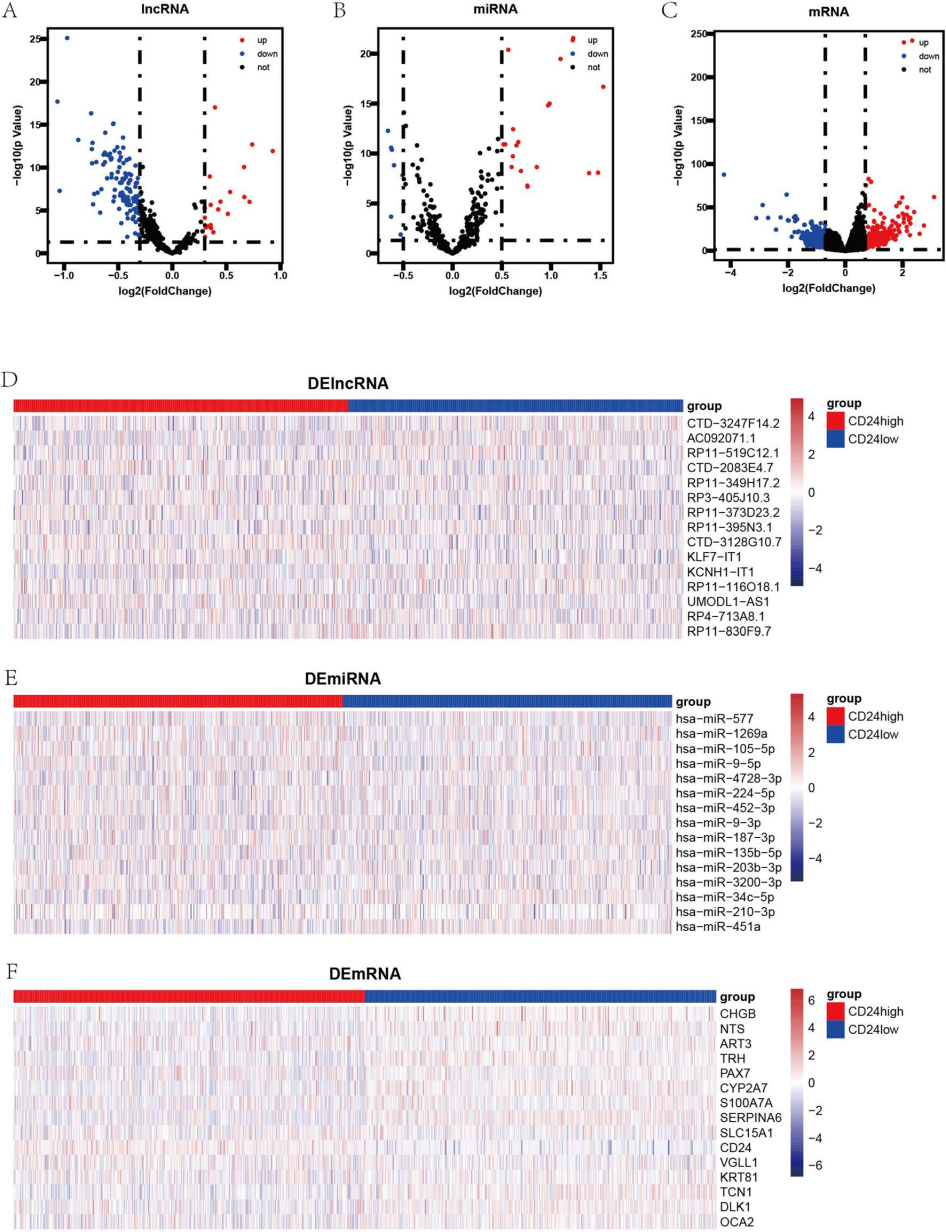
Differential expression analysis between tumor samples with high expression of CD24 and low expression of CD24. (**A**–**C**) Volcano maps of 132 DElncRNAs (p < 0.05 and |log2 fold change| > 0.3), 26 DEmiRNAs (p < 0.05 and |log2 fold change| > 0.5), 602 DEmRNAs (p < 0.05 and |log2 fold change| > 0.7). (**D**–**F**) Heatmaps of top 15 DElncRNAs, DEmiRNAs and DEmRNAs (sorted by p value).

According to the median of CD24 RNA expression, BRCA samples were divided into CD24^high^ and CD24^low^ groups, and we then performed difference analysis between the transcriptomic data of the two groups to identify the DEmRNAs, DElncRNAs and DEmiRNAs. We set *p* < 0.05 and |log2 fold change|> 0.7 as the DEmRNAs selection criteria, *p* < 0.05 and |log2 fold change|> 0.3 as the DElncRNAs selection criteria, and *p* < 0.05 and |log2 fold change|> 0.5 as DEmiRNAs selection criteria. In total, we selected 602 DEmRNAs (290 downregulated and 312 upregulated), 132 DElncRNAs (113 downregulated and 19 upregulated), and 26 DEmiRNAs (7 downregulated and 19 upregulated) from BRCA samples. The volcano plots exhibited the distribution of DElncRNAs, DEmiRNAs and DEmRNAs (Fig. 3A/B/C), and the heatmaps showed the RNA expression of 15 significant DE RNAs (Fig. 3D/E/F).

### Functional enrichment analysis of DEmRNAs

**Figure 4:**
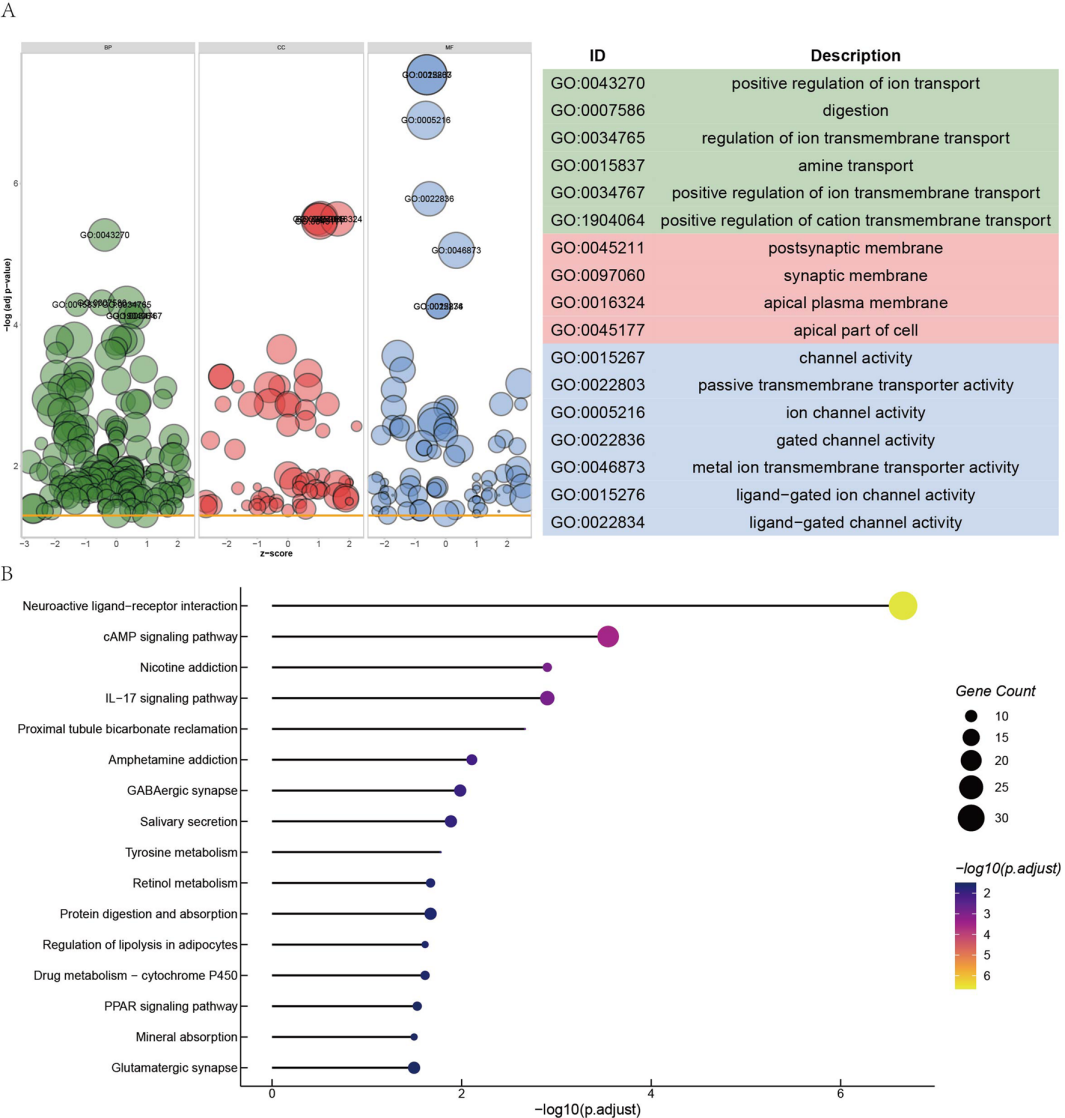
Functional enrichment analysis of DEmRNAs. (**A**) Annotation of MF, BP and CC for DEmRNAs. (**B**) KEGG pathway enrichment analysis of DEmRNAs.

To dig out the potential biological mechanisms related with CD24 involved in BRCA, the current study explored the function of CD24-associated DEmRNAs via Gene Ontology (GO) and Kyoto Encyclopedia of Genes and Genomes (KEGG) pathway analysis using ClusterProfiler R package. GO dot plot showed GO terms with gene counts > 3 and *p* value < 0.05 (Fig. 4A). The most enriched GO term in biological process (BP) was “positive regulation of ion transport digestion”, one in cellular component (CC) was “postsynaptic membrane”, and one in molecular function (MF) were “channel activity”. The lollipop chart exhibited total KEGG pathways with *p* value < 0.05 (Fig. 4B). Neuroactive ligand-receptor interaction was dominant pathway with highest gene counts (n = 34) and lowest p value (*p* = 2.2e7).

### Construction of the ceRNA network and identification of hub RNAs

**Figure 5:**
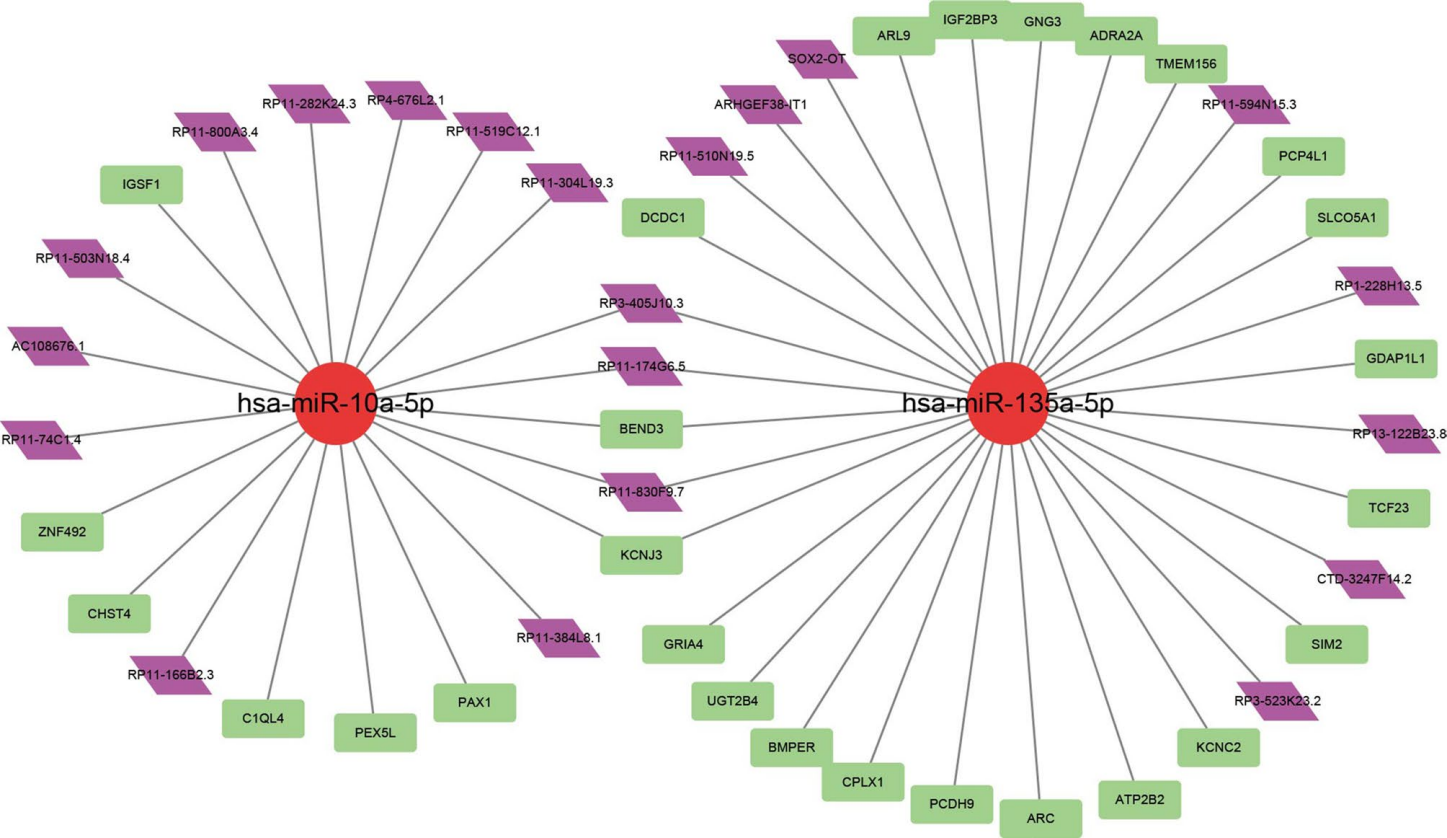
Construction of CD24-associated ceRNA network.

The current study took use of two databases to identify lncRNA-miRNA-mRNA ceRNA pairs. Finally, 24 lncRNA-miRNA interaction pairs and 29 miRNA-mRNA interaction pairs were identified. The ceRNA network (Fig. 5) which was constructed via Cytoscape 3.8, which included 21 DElncRNAs (6 upregulated and 15 downregulated), 2 DEmiRNAs (2 downregulated) and 29 DEmRNAs (12 upregulated and 17 downregulated).

**Figure 6:**
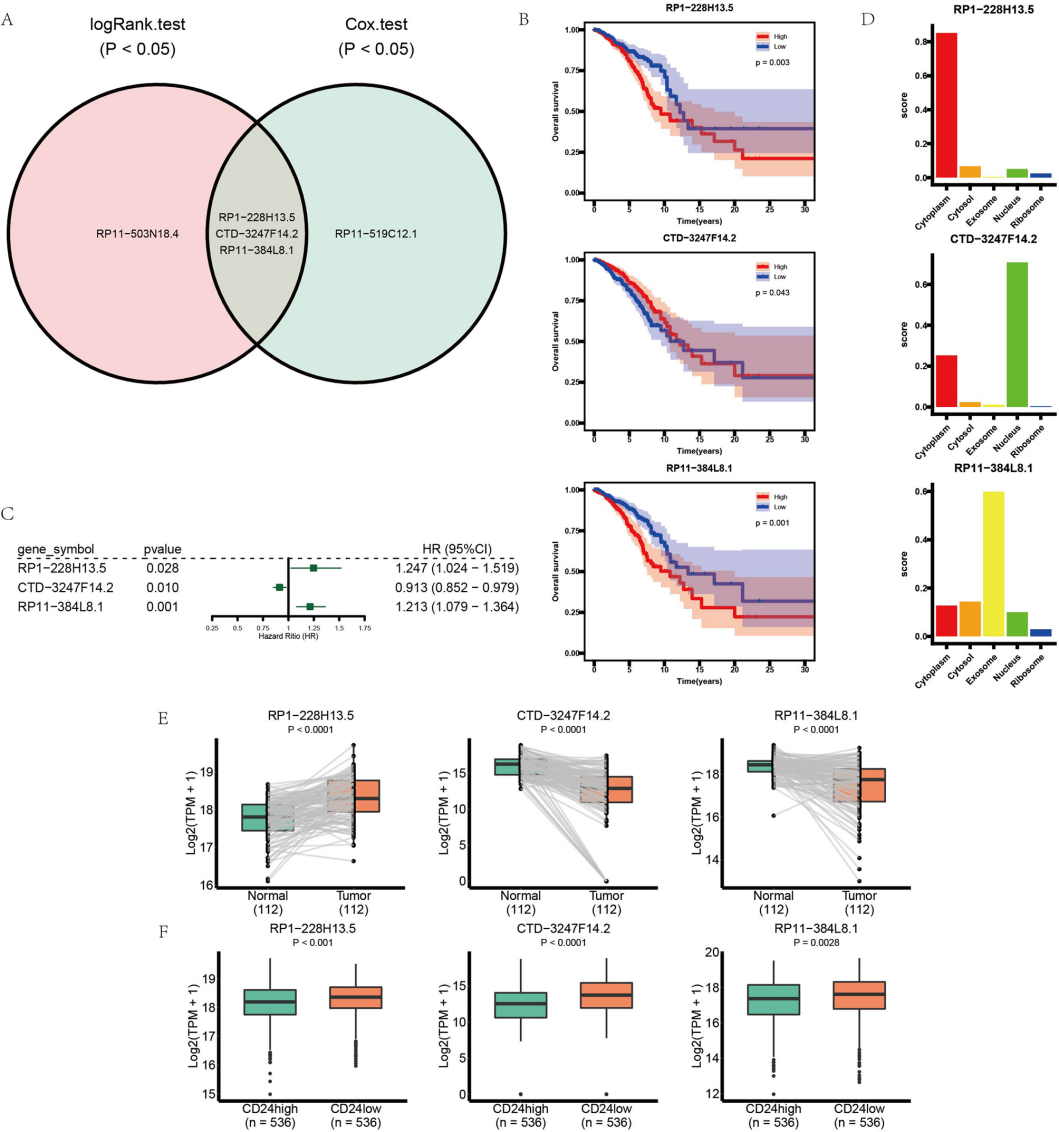
Comprehensive analysis of DElncRNAs. (**A**) Veen diagram of prognosis-related DElncRNAs in CD24-based ceRNA network. (**B**) Kaplan-Meier curve analysis of key DElncRNAs. (**C**) Univariate Cox analysis of key DElncRNAs. (**D**) Predicted results of subcellular localization of DElncRNAs through lncLocator web tool. (**E**) The expression levels of key DElncRNAs in tumor samples and paired adjacent normal samples. (**F**) The expression levels of key DElncRNAs in two groups: CD24high samples and CD24low samples.

Through survival analysis, three lncRNAs were identified with *P* < 0.05 (logRank test) and *P* < 0.05 (univariate Cox regression analysis) (Fig. 6A). According to the result of survival analysis with logRank test (Fig. 6B), patients with lower expression of RP1-228H13.5 (*P* = 0.003) and RP11-384L8.1 (*P* = 0.001) had better prognostic results, whereas those with lower expression of CTD-3247F14.2 had poor prognostic results. Univariate Cox regression analyses showed the similar result (Fig. 6C). RP1-228H13.5 (hazard ratio = 1.247, *P* = 0.028) and RP11-384L8.1 (hazard ratio = 1.217, *P* = 0.001) were identified as risk features, whereas CTD-3247F14.2 (hazard ratio = 0.913, *P* = 0.010) was identified as a favorable factor.

The current study predicted the location of three lncRNAs via lncLocator tool. The prediction results of LncLocator web (Fig. 6D) showed that RP1-228H13.5 was mainly located in cytoplasm, CTD-3247F14.2 mainly located in nucleus, and RP11-384L8.1 mainly located in exosome. Additionally, we verified different expression level of lncRNAs in 112 paired BRCA samples from TCGA database. RP1-228H13.5 was upregulated in tumor samples, whereas CTD-3247F14.2 and RP11-384L8.1 were downregulated in tumor samples (Fig. 6E). Figure 6F exhibited expression levels of 3 lncRNAs between CD24high and CD24low groups and there were obvious differences between two groups.

**Figure 7:**
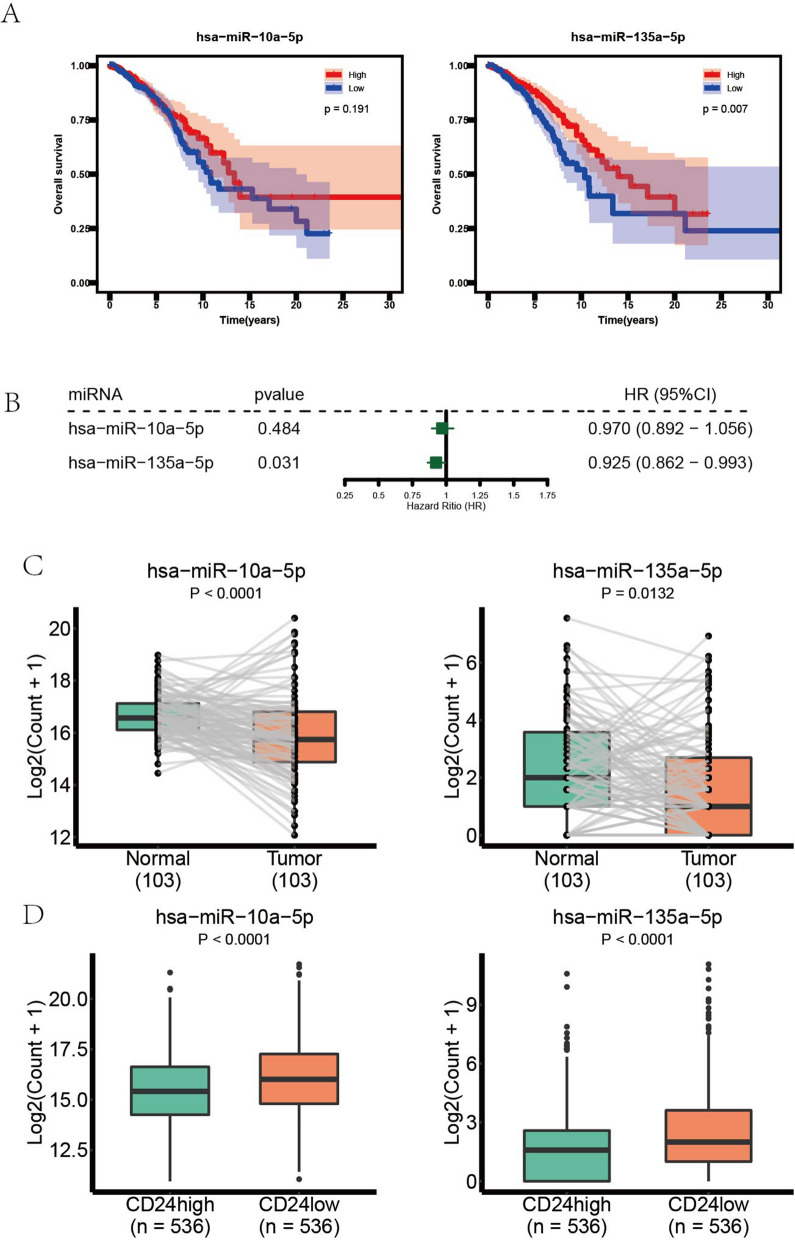
Comprehensive analysis of DEmiRNAs. (**A**) Kaplan-Meier curve analysis of DEmiRNAs. (**B**) Univariate Cox analysis of DEmiRNAs. (**C**) The expression levels of DEmiRNAs in tumor samples and paired adjacent normal samples. (**D**) The expression levels of DEmiRNAs in two groups: CD24high samples and CD24low samples.

Comprehensive analysis of the above indicated that RP1-228H13.5 was the hub lncRNA in the ceRNA network. Hsa-miR-135a-5p, the target of lncRNA RP1-228H13.5, was comprehensively analyzed. According to survival analysis, hsa-miR-135a-5p was identified as a protective factor (Fig. 7B) and the patients with higher expression level of hsa-miR-135a-5p had better results (Fig. 7A). Moreover, hsa-miR-135a-5p was downregulated in the tumor tissues compared with paired normal tissues (*P* = 0.0132) (Fig. 7C), and there was significant difference of expression level between CD24^high^ and CD24^low^ groups (Fig. 7D). In contrast, another miRNA, hsa-miR-10a-5p, seemed less important. There was no significant relationship between hsa-miR-10a-5p and prognostic results of patients with BRCA, although there was significant difference in differential analysis of hsa-miR-10a-5p between paired normal and tumor samples (Fig. 7C) as well as between the groups with high CD24 expression and low CD24 expression (Fig. 7D).

**Figure 8:**
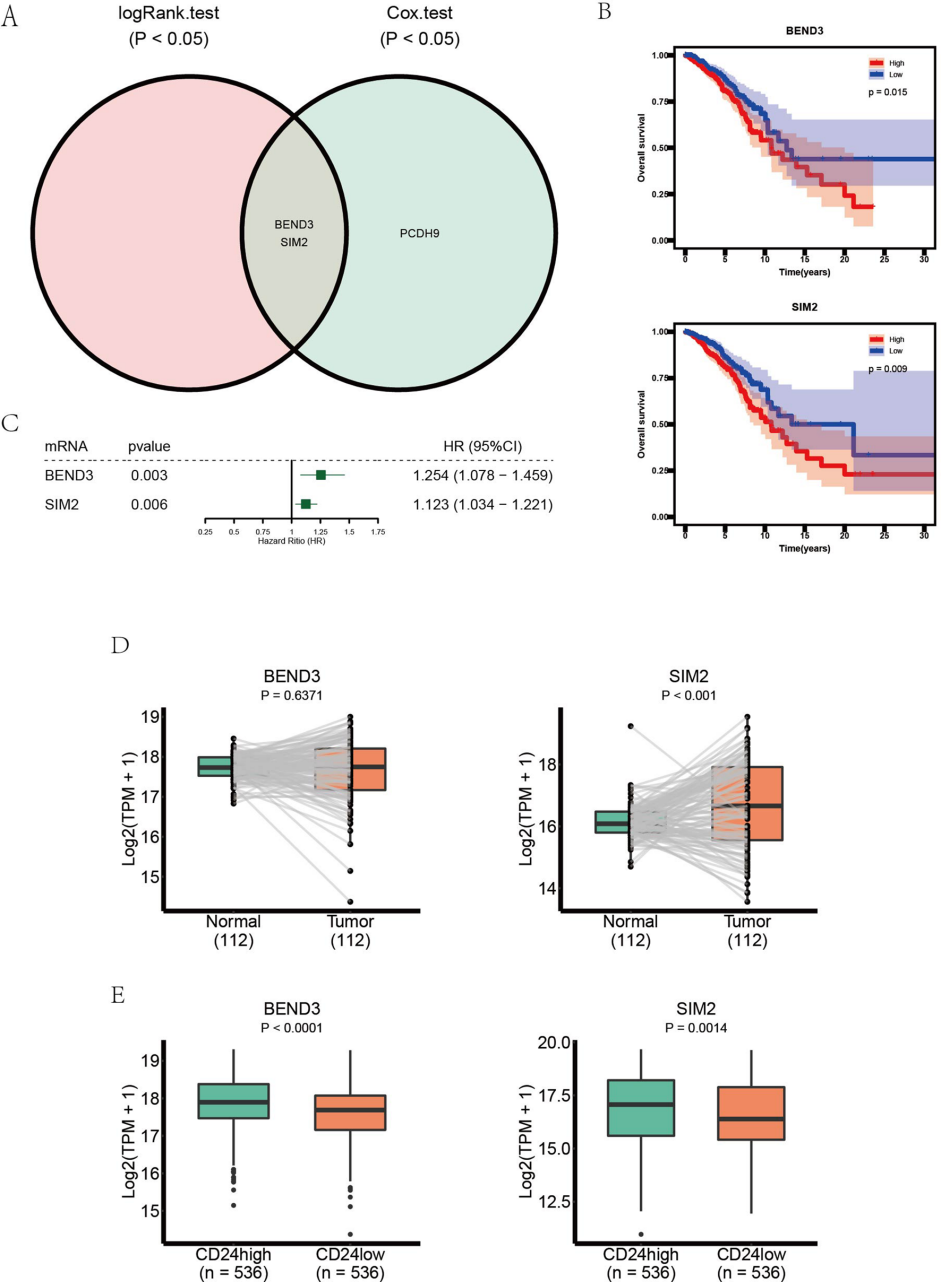
Comprehensive analysis of DEmRNAs. (**A**) Veen diagram of prognosis-related DEmRNAs in CD24-based ceRNA network. (**B**) Kaplan-Meier curve analysis of key DEmRNAs. (**C**) Univariate COX analysis of key DEmRNAs. (**D**) The expression levels of key DEmRNAs in tumor samples and paired adjacent normal samples. (**E**) The expression levels of key DEmRNAs in two groups: CD24high samples and CD24low samples.

Among the target mRNAs of hsa-miR-135a-5p, the current study identified that BEND3 and SIM2 were hub mRNAs. We estimated the prognostic value of target genes via Kaplan–Meier curves (logRank test) and univariate Cox regression analysis. As shown in Fig. 8A, BEND3 and SIM2 were the mRNAs with *P* < 0.05 in both logRank test and univariate Cox regression analysis. Kaplan–Meier curve showed that the patients of BRCA with lower expression level of BEND3 (LogRank test, *P* = 0.015) and SIM2 (LogRank test, P = 0,009) had better prognostic results (Fig. 8B). And forest plot exhibited that BEND3 and SIM2 served as risk factors in the development of BRCA (*P* = 0.003 and *P* = 0.006 respectively) (Fig. 8C). The expression level of BEND3 in paired tumor and tissue samples had no significant difference (Fig. 8D), whereas the difference of expression level of BEND3 between groups of CD24^high^ and CD24^low^ was significant (Fig. 8E). SIM2 was upregulated in the paired tumor tissues compared with paired normal tissues (*P* < 0.001) (Fig. 8D), and was downregulated in the CD24^low^ group compared with CD24^high^ group (*P* = 0.0014) (Fig. 8E).

**Figure 9:**
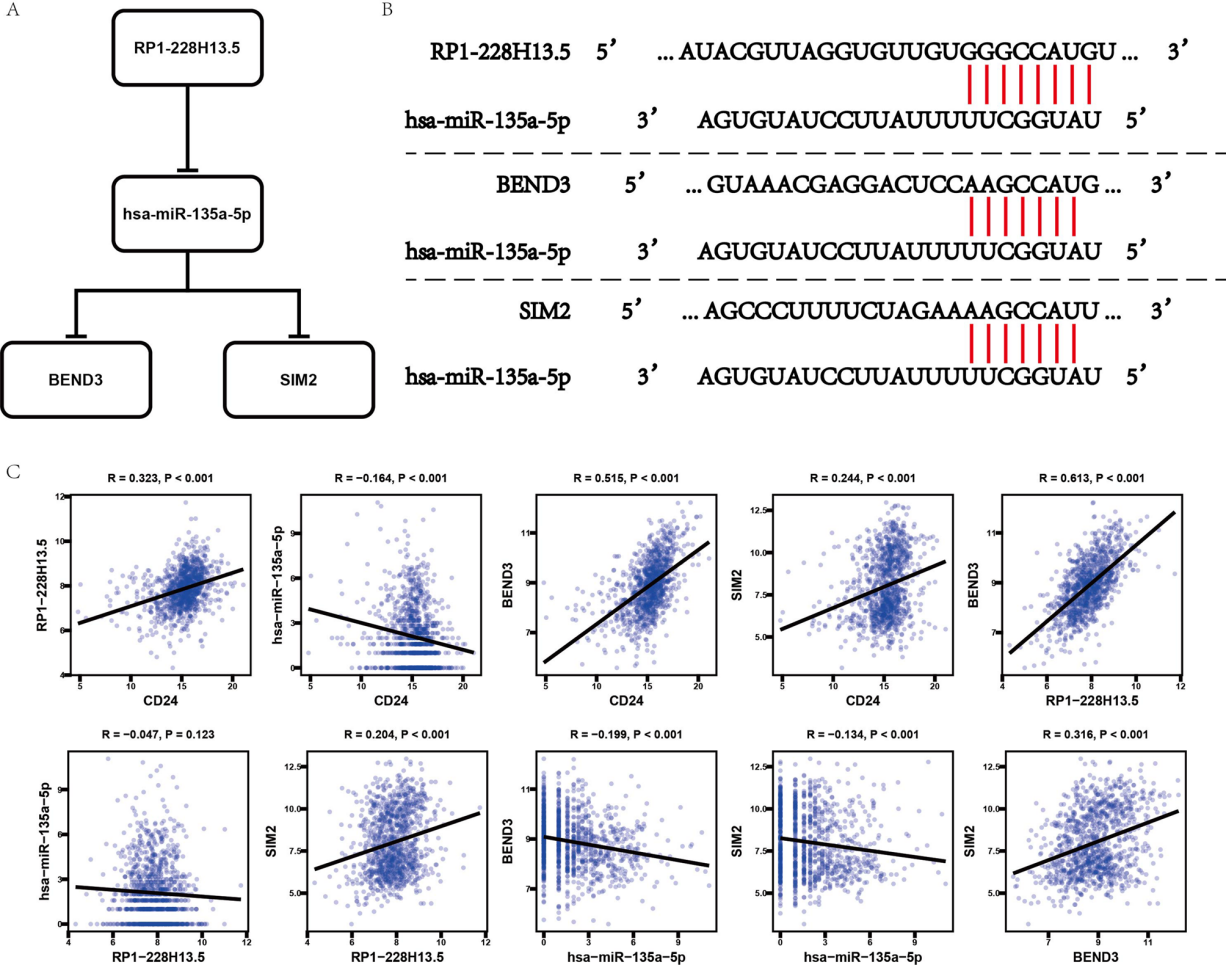
The identification significant ceRNA axis. (**A**) Schematic diagram of significant ceRNA axis. (**B**) The sequence of significant genes in ceRNA network. (**C**) Co-expression analysis of significant genes in ceRNA network.

To sum up the above analysis, we identified that RP1-228H13.5, hsa-miR-135a-5p, BEND3 and SIM2 composed hub ceRNA pair which was highly associated with CD24 (Fig. 9A). The binding sites of RP1-228H13.5, BEND3, SIM2 and hsa-miR-135a-5p were shown in Fig. 9B. Then, we performed expression correlation analysis for ceRNA network (Fig. 9C). It was confirmed that RP1-228H13.5 was positively correlated with BEND3 (R = 0.613, *P* < 0.001) and SIM2 (R = 0.204, *P* < 0.001). What’s more, CD24 was found highly correlated with genes of ceRNA network as well.

### Clinical relevance of ceRNA network in BRCA patients

**Figure 10:**
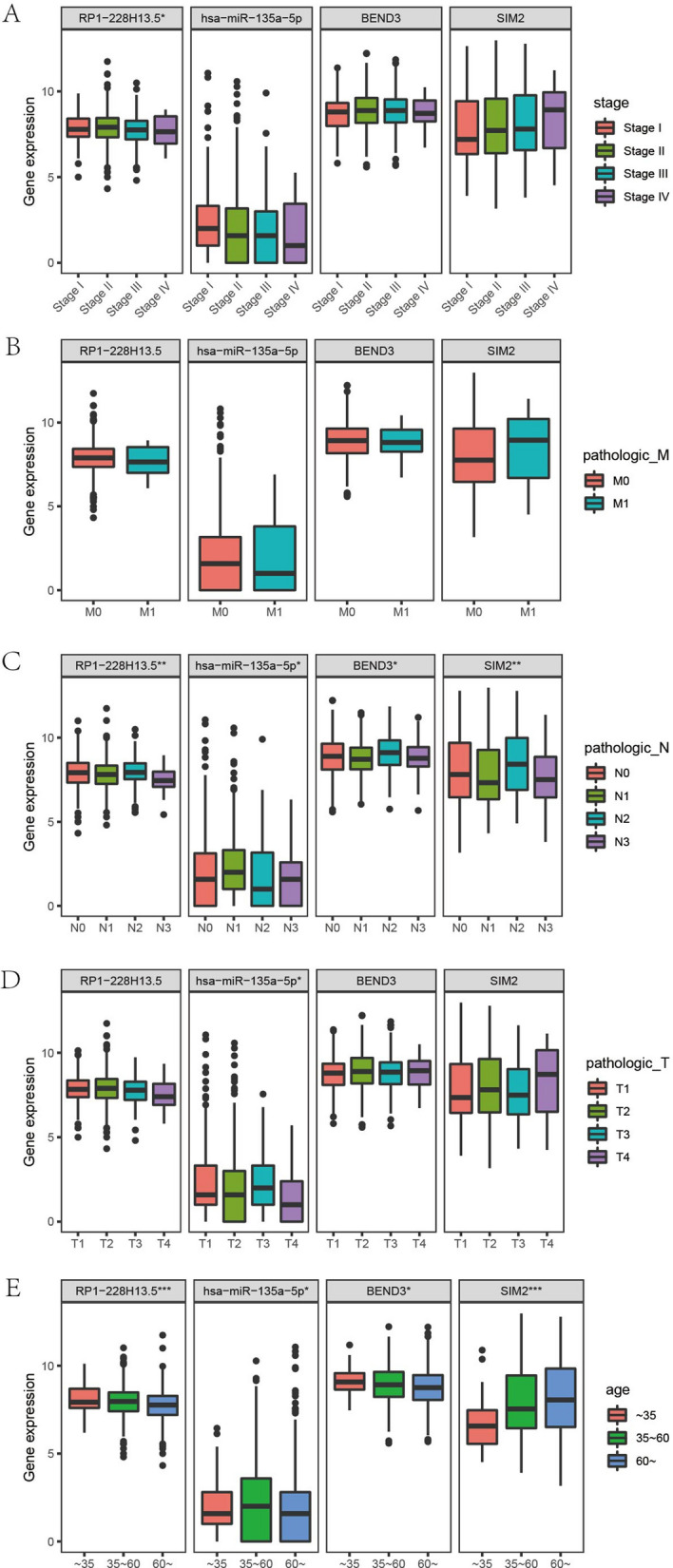
Clinical correlation analysis of key genes in ceRNA network. (**A**–**E**) Correlation analysis between key genes and clinical stage, pathological metastasis stage, pathological N stage, pathological T stage and age at initial pathologic diagnosis.

To further explore clinical relevance of ceRNA network, the current study analyzed the relationships between hub RNAs (RP1-228H13.5, hsa-miR135a-5p, BEND3 and SIM2) and various clinical features, such as age, clinical stage, tumor status, distant metastasis and lymph code metastasis. The ceRNA network was found highly related with lymph code metastasis (Fig. 10C) and patients’ age (Fig. 10E), but was rarely related with other clinical information (clinical stage, M/T stage) (Fig. 10A/B/D).

### Relationship between immune infiltration and expression levels of BEND3/SIM2

**Figure 11:**
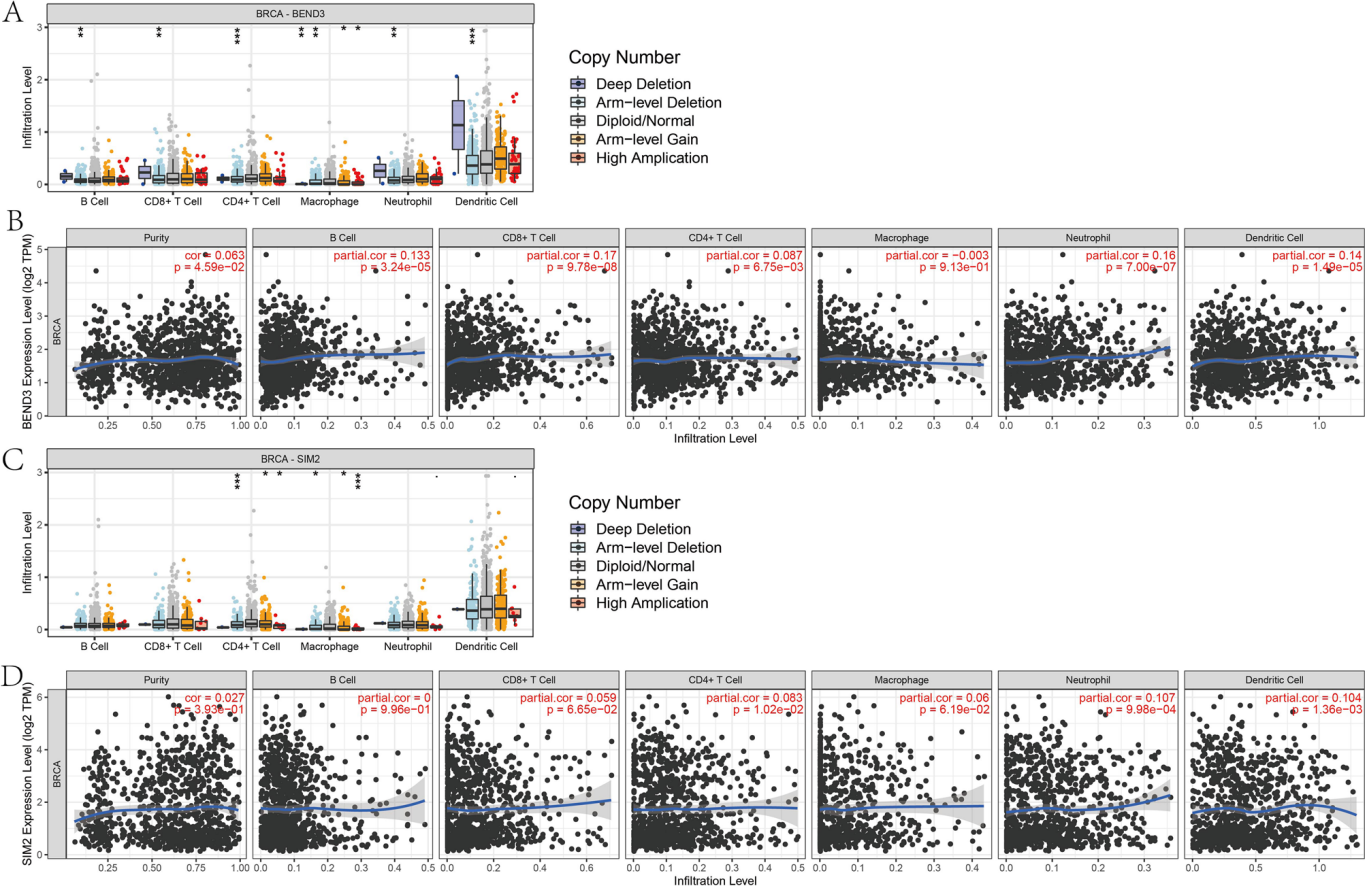
Comprehensive analysis of BEND3 (**A**, **B**) and SIM2 (**C**, **D**) and tumor immune microenvironment based on the TIMER database.

Immune infiltration is a mature factor involved in the initiation, development and prognostic of tumors. Hence, we further explored the relationships between immune infiltration and expression of BEND3/SIM2 via TIMER database. As shown in Fig. 11A/C, several immune cells are correlated with the altered copy number of BEND3/SIM2. Figure 11B/D exhibited the relationship between BEND3/SIM2 and tumor purity, B cells, CD8 + T cell, CD4 + T cell, Macrophage, Neutrophil and Dendritic cell. BEND3 was correlated with CD8 + T cell to a certain extent (Partial.cor = 0.17, *p* = 9.78e−08), whereas SIM2 had no notable correlation with these immune cells.

**Figure 12:**
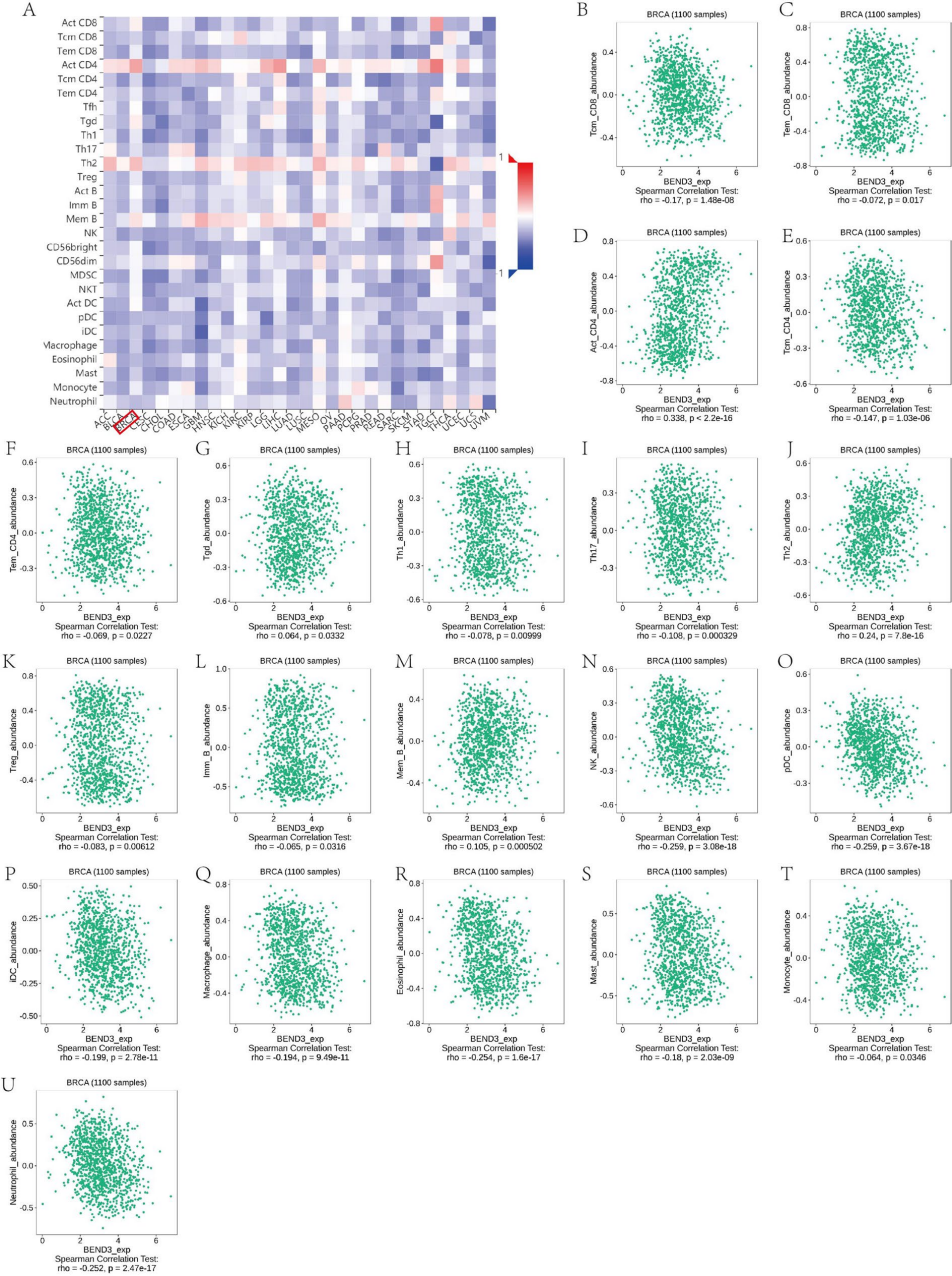
Comprehensive analysis of BEND3 and tumor immune microenvironment based on the TISIDB database. (**A**) The correlations of BEND3 with immune cells across cancers. (**B**–**U**) The significant correlations between BEND3 and immune cells in BRCA.

**Figure 13:**
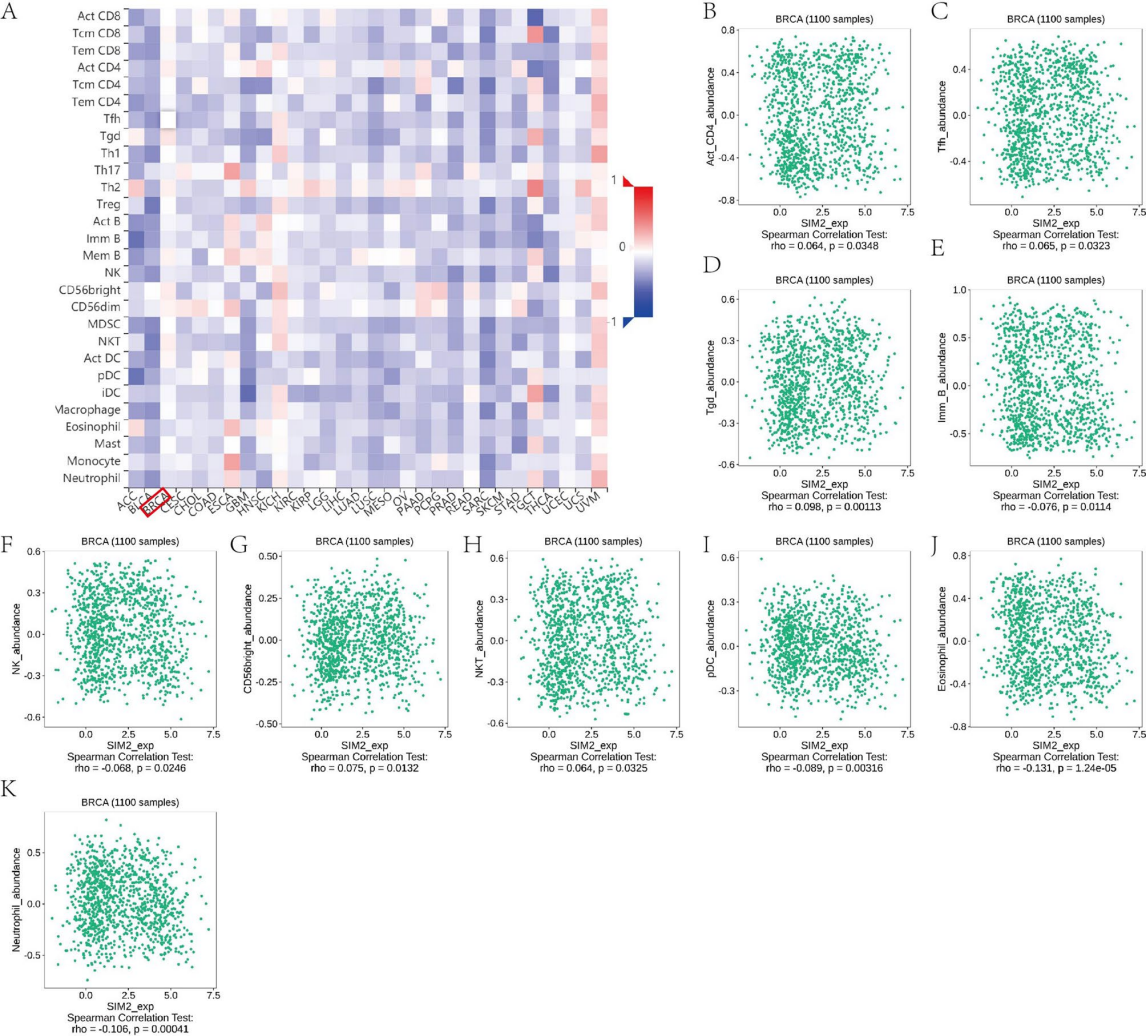
Comprehensive analysis of SIM2 and tumor immune microenvironment based on the TISIDB database. (**A**) The correlations of SIM2 with immune cells across cancers. (**B**–**K**) The significant correlations between SIM2 and immune cells in BRCA.

Then, based on TISIDB database, we explored the relationship between BEND3/SIM2 expression and abundance of 28 tumor-infiltrating lymphocytes (TILs) (Figs. 12, 13). We found that BEND3 had notable correlation with Act CD4 + T cell (r = 0.338, *P* < 2.2e−16), Th2 cell (r = 0.24, *P* = 7.8e−16), NK cell (r = − 0.259, *P* = 3.08e−18), pDC, Eosinophil cell (− 0.254, *P* = 1.6e−17) and Neutrophil cell (r = − 0.252, *P* = 2.47e−17). On the contrast, SIM2 had no notable correlation with TILs.

**Figure 14:**
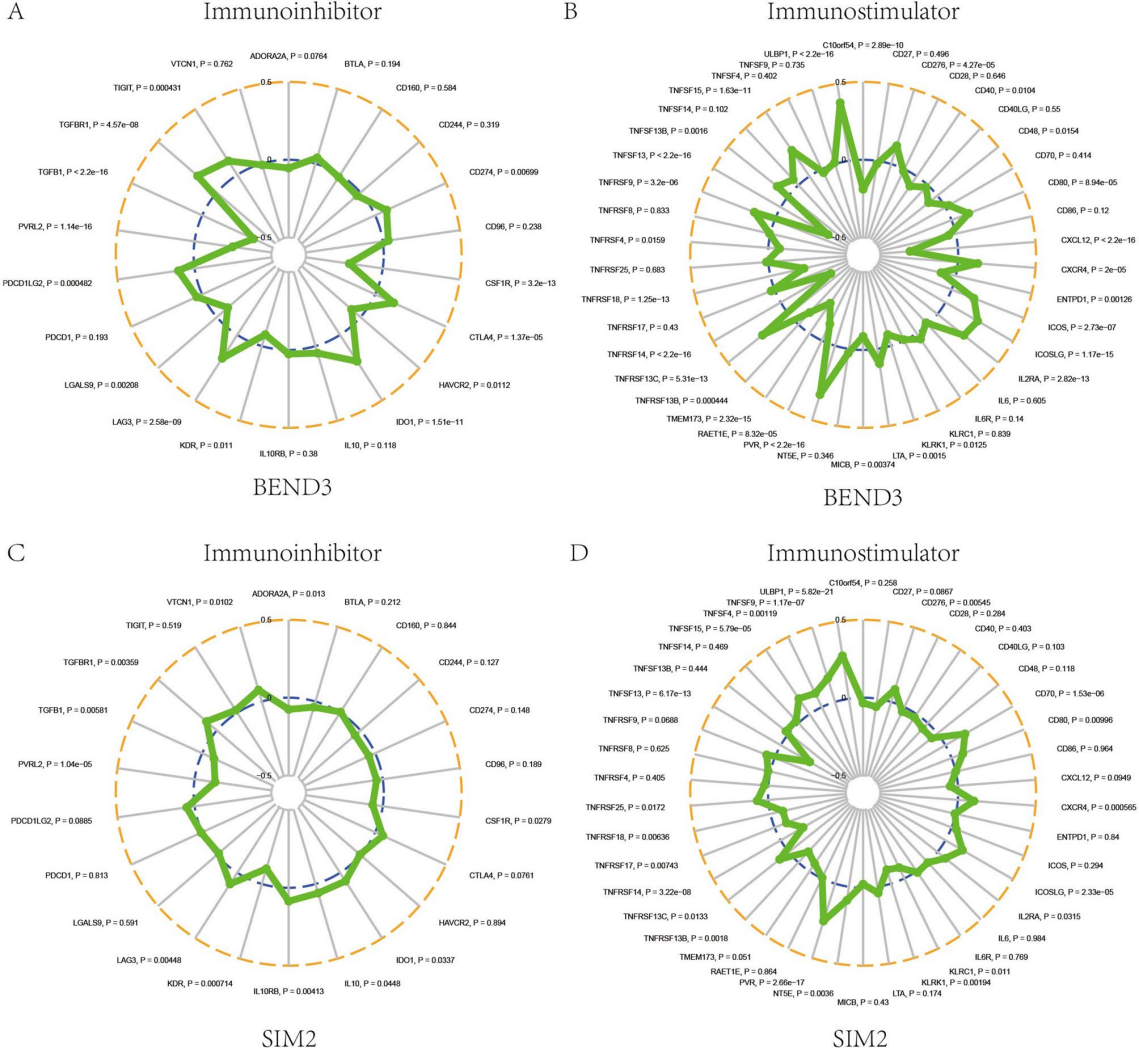
Comprehensive analysis of BEND3 and SIM2 and various immune checkpoints based on the TISIDB database. (**A**–**B**) The correlations of BEND3 with immune inhibitor genes and immune stimulator genes. (**C**–**D**) The correlations of SIM2 with immune inhibitor genes and immune stimulator genes.

An increasing number of evidences has proved that immune checkpoint inhibitor (ICI) is a significant immunotherapy strategy with great prospects. Through searching in TISIDB database, we got the correlations between BEND3/SIM2 and over 40 common immune checkpoint genes in BRCA (Fig. 14). We found that BEND3 had highly negative correlation with TGFβ1 (r = − 0.363, *P* < 2.2e−16), whereas showed significantly positive correlation with PVR (r = 0.329, *P* < 2.2e−16) and ULBP1 (r = 0.377, *P* < 2.2e−16). SIM2 exhibited significantly positive correlation with PVR (r = 0.252, *P* = 2.66e−17) and ULBP1 (r = 0.279, *P* = 5.82e−21).

### Relationship between methylation and expression of BEND3/SIM2

**Figure 15:**
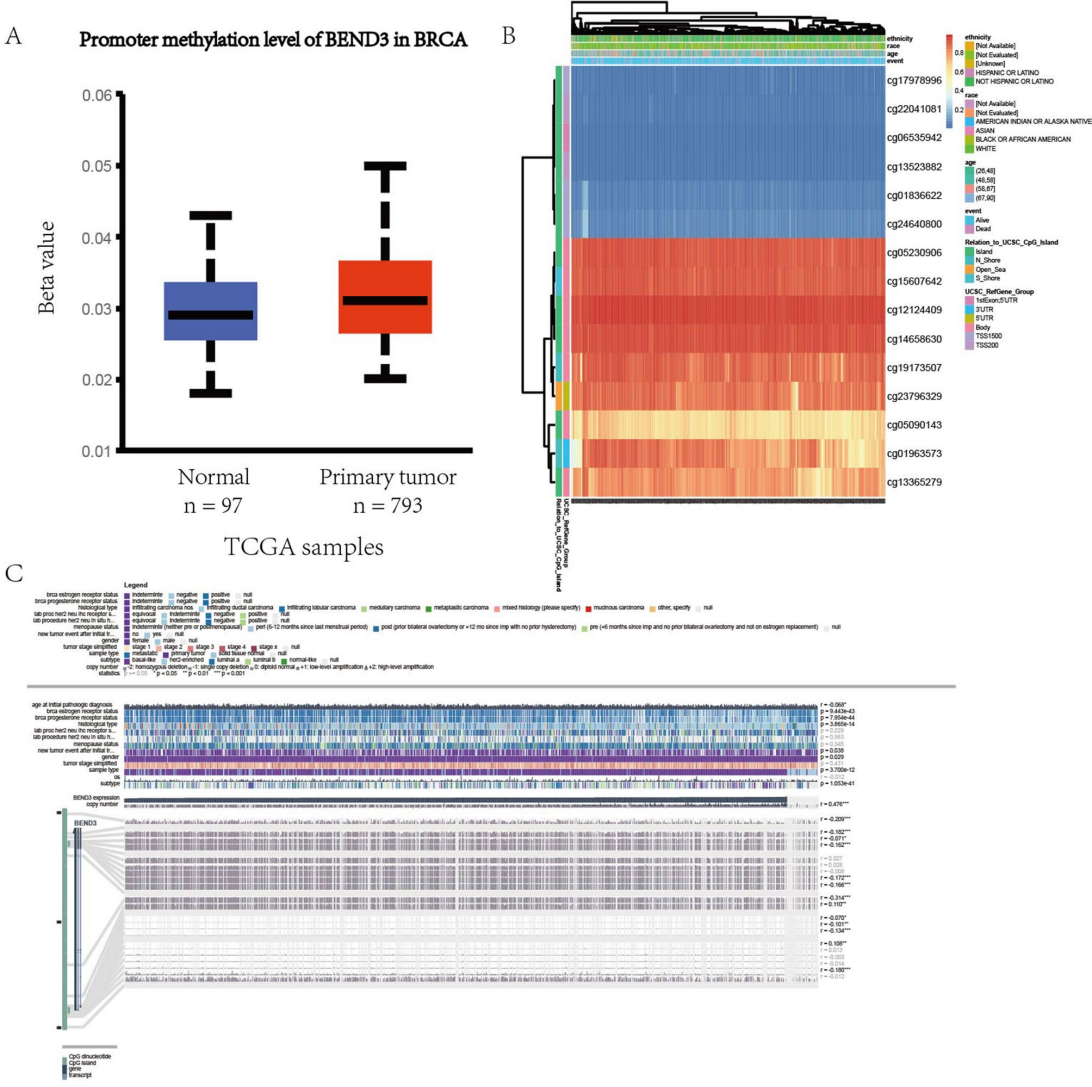
Analysis of BEND3 and methylation. (**A**) The promoter methylation level of BEND3 in BRCA. (**B**) The correlations between methylation sites of BEND3 and clinical features in BRCA. (**C**) The methylation information of CpG islands of BEND3.

**Figure 16:**
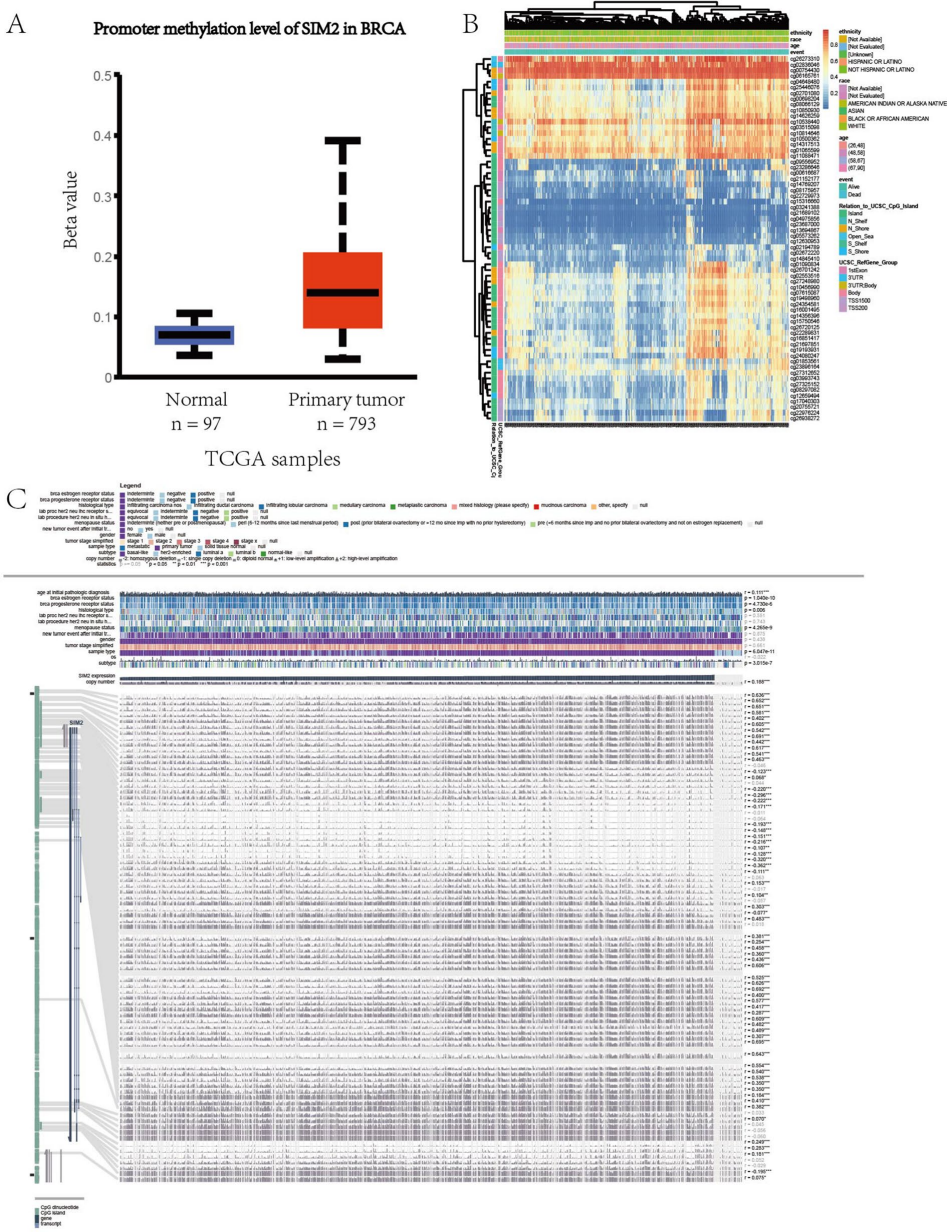
Analysis of SIM2 and methylation. (**A**) The promoter methylation level of SIM2 in BRCA. (**B**) The correlations between methylation sites of SIM2 and clinical features in BRCA. (**C**) The methylation information of CpG islands of SIM2.

In order to further explore the dysregulated mechanisms of BEND3/SIM2, the current study performed an analysis for their methylation status. We found that there was no significant difference between the promoter methylation level of BEND3 in normal tissues and primary tumor tissues (Fig. 15A). However, the promoter methylation level of SIM2 in BRCA tissues was significantly higher than normal tissues (Fig. 16A). The methylation of BEND3 occurred on some CpG islands, including cg23339693, cg23796329 (r = − 0.209, − 0.314, respectively) (Fig. 15C). In the meanwhile, the methylation of SIM2 occurred on a great number of sites (Fig. 16C). Figures 15B and 16B, respectively, showed the correlations of methylation sites of BEND3 and SIM2 with BRCA clinical factors.

## Discussion

Breast cancer, the most common malignancy in women, is considered potentially curable in early stage, and molecular targeted therapy has been confirmed significantly helpful in BRCA patients. However, only a minority of patients appear to respond. Hence, it is a top priority to find new therapy targets and novel biomarkers. Many studies have reported that ceRNA mechanism is involved in the initiation and development of breast cancer and has a potential to work as therapy targets or prediction factors^51^.

CD24 is a membrane protein overexpressed in many solid tumors, and has a role in promoting immune evasive via interacting with sialic-acid-binding Ig-like lectin 10 (Siglec-10), which is expressed by tumor-associated macrophages^52^. In various cancers, CD24 expressed by cancer cells is described as “do not eat me” signal, which is identified associated with tumor growth in vitro and in vivo. What’s more, CD24 also serves as cancer stemness marker^53^. However, to our best knowledge, the ceRNA network associated with CD24 remains unknown. In this study, we downloaded RNAseq data of BRCA from TCGA database, divided them into CD24^high^ and CD24^low^ groups based on the median expression level of CD24, and performed a difference analysis. Then we constructed a ceRNA network using 21 DElncRNAs, 2 DEmiRNAs and 29 DEmRNAs.

lncRNA is dominant in the ceRNA triple regulatory network. Accordingly, we first analyzed DElncRNAs. Prognostic analysis reflected that RP1-228H13.5, CTD-3247F14.2 and RP11-384L8.1 were the prognostic signatures, among which RP1-228H13.5 was the only lncRNA which was predicted located in cytoplasm. What’s more, RP1-228H13.5 was upregulated in BRCA tumor tissues compared with paired adjacent tissues, and also had higher RNA expression levels in CD24^low^ group compared with CD24^high^ group. RP1-228H13.5 has been studied in some cancers. A study reported that RP1-228H13.5, referred in that study as CRAL, is downregulated in cisplatin-resistance in gastric cancer (GC) cells and contributes to cisplatin-resistant in GC cells via impairing cisplatin-induced DNA damage as well as cell apoptosis through the miR-505/CYLD/AKT axis^54^. Another study as well reported that the RNA expression level of RP1-228H13.5 is associated with HCC patients’ overall and recurrence-free survival through performing a data mining using RNA sequencing data from TCGA and two microarray datasets from GEO^55^. However, too little work has been devoted to the role of RP1-228H13.5 in breast cancer.

According to the ceRNA hypothesis, miRNA is the bridge connecting lncRNA and mRNA in various biological processes. In our study, hsa-miR-135a-5p was found downregulated in BRCA tissues and was identified as a protective factor. The role of hsa-miR-135a-5p has been explored in various cancers, including lung cancer, hepatocarcinogenesis, tongue squamous cell carcinoma, and ovarian cancer^56–59^. Nevertheless, its function is different in different tumors. For instance, hsa-miR-135a-5p is upregulated in NSCLC tissues and its loss can restrain lung cancer via interacting with LOXL4^56^. On the contrast, hsa-miR-135a-5p is reported downregulated in ovarian cancer tissues and functions as a tumor suppressor in ovarian cancer via binding CCR2^59^. Similar with RP1-228H13.5, the function of hsa-miR-135a-5p in BRCA remains largely under-explored.

Messenger RNA is able to perform specific biological activities via coding proteins. CD24-associated mRNAs were performed GO enrichment analysis and KEGG pathway analysis, and were found highly correlated with channel activity.

BEND3 is a transcriptional repressor which associated with the nucleolar remodeling complex (NoRC) and plays a key role in repressing rDNA transcription. In GO database, BEND3 is annotated by “rDNA heterochromatin assembly”^60,61^. It is reported that BEND3 is associated with NuRD, NoRC and PRC2 complexes, and plays an important role in the transcription regulatory^62^. Recently, in acute myeloid leukemia, BEND3 is identified as a key gene whose deletion results in resistance to TAK-243, a first-in-class inhibitor of ubiquitin-like modifier activating enzyme 1, via regulating breast cancer resistance protein (BCRP)^63^. The impact of BEND3 in breast cancer has been observed as well. In 2017, a study has confirmed that BEND3 is dysregulated in Basal-like breast cancer^64^. Moreover, BEND3 is identified upregulated in BRCA cancers with visceral organ metastasis^65^. However, the molecular mechanism of BEND3 in the initiation, development of breast cancer remains unclear. Although our study revealed that methylation had rare influence on BEND3 in BRCA, BEND3 was reported having a huge impact on methylation level of genes via binding CpG island. Recently, researchers found that BEND3 occupies active (H3K4me3) and bivalent promoters (H3K4me3 and H3K27me3), and BEND3-bound regions have lower CpG methylation level compared with the unbound sites^66^. Although gene transcription level is usually considered to be negatively correlated with DNA methylation level of promoter in the past, more and more studies support that the high methylation level of promoter can induce gene expression. For example, CD24, the promoter of which exhibited high DNA methylation level, was confirmed overexpressed^67^. Given complex epigenetic regulatory mechanisms, we guess that BEND3 can promote CD24 expression via hypermethylating its promoter, which deserves further research. BEND3 also exhibited high correlation with the infiltration levels of B cell, CD8 + T cell, Neutrophil and Dendritic cell. In addition, the current study explored the correlation between BEND3 and immune check points. We found that BEND3 was highly negatively associated with TGFβ1, TNFSF13, TNFRSF14, CXCL12, whereas was significantly positively correlated with ULBP1, PVR, TNFRSF13c. In summary, BEND3 is a significant gene highly associated with CD24 in breast cancer, and serves as a potential therapeutic target.

SIM2 encodes a transcription factor that is a master regulator of neurogenesis. SIM2 firstly is well-known as a Down’s syndrome critical locus gene, whereas then is found associated with some solid tumors, for which SIM2 is a promising novel treatment target^68^. As a transcription factor, SIM2 is involved in tumors via diverse molecular mechanisms. For example, in pancreatic cancer, the silence of SIM2-s (the short form of SIM2) significantly induces CAPAN-1 cell death via apoptosis^69^. SIM2 is identified overexpressed in prostate cancer tissues as well, and works as a potential target for the immunotherapy of prostate cancer^70^. Many studies reported that SIM2 plays a significant role in breast cancer. KC Scribner et al. confirmed that SIM2s regulate the progression of primary breast ductal carcinoma to invasive breast cancer through epithelial mesenchymal transition (EMT)^71^. A novel role of SIM2s is demonstrated by a study in 2019, in which SIM2s is found to attenuate the expression of COX2/PTGS2 via mediating NFκB signaling^72^. In our study, we found the promoter methylation level of SIM2 in primary tumor was upregulated. However, this methylation level still belongs to hypomethylated level, which could not explain the change of SIM2. In addition, many CpG islands of SIM2 were identified. Combined with previous studies, we hypothesize that the methylation of SIM2 is a potential key regulation mechanism in the procession of breast cancer. We also found that SIM2 gene copy numbers were associated with the infiltration level of CD4 + T cells and macrophages. Some immune cells also showed associations with the expression level of SIM2.

Although we explored the role of CD24-related ceRNA regulator network in breast cancer from multiple dimensions, there were limitations to be considered in this study. First, the dataset from TCGA was the only data analyzed in this study, and more datasets from different sources needed to be included in the analysis. Second, key ceRNA interactions need to be validated by experiments in the further study. Finally, the roles of BEND3 and SIM2 in tumor immune microenvironment in breast cancer was obtained using online web tools, which requires more experimental validations.

## Conclusion

Using the RNA sequence data and clinical information form TCGA database, we established a CD24-asssociated ceRNA network (RP1-228H13.5/miR-135a-5p/BEND3 and SIM2) and made a comprehensive analysis via a diversity of bioinformatic tools, which shed a new light on the mechanism of BRCA. These novel critical biomarkers were significantly related with prognosis, and had an impact on tumor immune microenvironment (Supplementary information).

## Supplementary Information

Supplementary Information.

## Abbreviations

BRCA: Breast invasive carcinoma
TNBC: Triple-negative breast cancer
ncRNA: Non-coding RNA
circRNA: NcRNAs comprise circular RNA
lncNRA: Long non-coding NRA
miRNA: MicroNRA
piRNA: PIWI interacting RNA
TCGA: The Cancer Genome Atlas
DEGs: Differentially expressed genes
DElncRNAs: Differentially expressed lncRNAs
DEmiRNAs: Differentially expressed miRNAs
DEmRNAs: Differentially expressed mRNAs
GO: Gene Ontology
BP: Biological process
CC: Cellular component
MF: Molecular function
KEGG: Kyoto Encyclopedia of Genes and Genomes
GC: Gastric cancer
TILs: Tumor-infiltrating lymphocytes
NoRC: Nucleolar remodeling complex
BCRP: Breast cancer resistance protein
EMT: Epithelial mesenchymal transition
